# Screening for Interacting Proteins with Peptide Biomarker of Blood–Brain Barrier Alteration under Inflammatory Conditions

**DOI:** 10.3390/ijms22094725

**Published:** 2021-04-29

**Authors:** Karina Vargas-Sanchez, Monica Losada-Barragán, Maria Mogilevskaya, Susana Novoa-Herrán, Yehidi Medina, Cristian Buendía-Atencio, Vaneza Lorett-Velásquez, Jessica Martínez-Bernal, Rodrigo E. Gonzalez-Reyes, David Ramírez, Klaus G. Petry

**Affiliations:** 1 Grupo de Neurociencia Translacional, Facultad de Medicina, Universidad de los Andes, Bogotá 111711, Colombia; 2 Grupo de Biología Celular y Funcional e Ingeniería de Moléculas, Departamento de Biología, Universidad Antonio Nariño, Bogotá 110231, Colombia; monica.losada@uan.edu.co (M.L.-B.); ymedina17@uan.edu.co (Y.M.); 3 Grupo de Investigación GINIC-HUS, Universidad ECCI, Bogotá 111311, Colombia; mmogilevskaya@ecci.edu.co; 4 Grupo de Investigación en Hormonas (Hormone Research Laboratory), Departamento de Química, Universidad Nacional de Colombia, Bogotá 111321, Colombia; ssnovoah@unal.edu.co or; 5 Grupo de Fisiología Molecular, Subdirección de Investigación Científica y Tecnológica, Instituto Nacional de Salud, Bogotá 111321, Colombia; 6 Grupo de Investigación en Modelado y Computación Científica, Departamento de Química, Universidad Antonio Nariño, Bogotá 110231, Colombia; c.buendia@uan.edu.co; 7 Facultad de Medicina y Ciencias de la Salud, Universidad Militar Nueva Granada, Bogotá 110231, Colombia; vaneza.lorett@unimilitar.edu.co (V.L.-V.); jam1042@scarletmail.rutgers.edu (J.M.-B.); 8 Grupo de Investigación en Neurociencias (NeURos), Centro de Neurociencia Neurovitae-UR, Escuela de Medicina y Ciencias de la Salud, Universidad del Rosario, Bogotá 111711, Colombia; rodrigo.gonzalez@urosario.edu.co; 9 Instituto de Ciencias Biomédicas, Facultad de Ciencias de la Salud, Universidad Autónoma de Chile, El llano Subercaseaux 2801, Santiago 8900000, Chile; david.ramirez@uautonoma.cl; 10 INSERM U1049 and U1029 Neuroinflammation and Angiogenesis Group, Bordeaux University, F33000 Bordeaux, France; klaus.petry@inserm.fr

**Keywords:** peptide biomarker, neuroinflammation, blood–brain barrier, laminin subunit beta-1, endothelium

## Abstract

Neurodegenerative diseases are characterized by increased permeability of the blood–brain barrier (BBB) due to alterations in cellular and structural components of the neurovascular unit, particularly in association with neuroinflammation. A previous screening study of peptide ligands to identify molecular alterations of the BBB in neuroinflammation by phage-display, revealed that phage clone 88 presented specific binding affinity to endothelial cells under inflammatory conditions in vivo and in vitro. Here, we aimed to identify the possible target receptor of the peptide ligand 88 expressed under inflammatory conditions. A cross-link test between phage-peptide-88 with IL-1β-stimulated human hCMEC cells, followed by mass spectrometry analysis, was used to identify the target of peptide-88. We modeled the epitope–receptor molecular interaction between peptide-88 and its target by using docking simulations. Three proteins were selected as potential target candidates and tested in enzyme-linked immunosorbent assays with peptide-88: fibronectin, laminin subunit α5 and laminin subunit β-1. Among them, only laminin subunit β-1 presented measurable interaction with peptide-88. Peptide-88 showed specific interaction with laminin subunit β-1, highlighting its importance as a potential biomarker of the laminin changes that may occur at the BBB endothelial cells under pathological inflammation conditions.

## 1. Introduction

The breakdown of the blood–brain barrier (BBB) and its associated increased permeability is an important feature of neurodegenerative diseases [1]. Nevertheless, the BBB does not act as an isolated system, as it performs its functions within a neurovascular unit (NVU), which includes cellular components such as endothelial cells, pericytes, neurons, astrocytes and microglia [2], as well as structures of the extracellular matrix (ECM) [3]. Astrocytes, in particular, due to their special cellular organization, are crucial to preserve functional aspects of both the BBB and the NVU. Astrocytes are the main regulators of brain energetic metabolism, providing neurons with necessary lactate and other sources of energy [4]. In addition, astrocytes are deeply involved in synaptic processes, integrating the tripartite synapse together with the presynaptic and postsynaptic neurons [5]. Therefore, alterations in any of these structural components could cause BBB disruption and facilitate the infiltration of peripheral immune cells into the brain parenchyma, leading to the development of abnormal neuroinflammatory reactions [6].

Brain endothelial cells are of particular interest to neuroscience research given their role in restricting the passage of harmful substances into the central nervous system (CNS) [7]. Complete BBB disruption has been shown to be preceded by inflammatory activity under pathological circumstances. This occurs when peripheral immune cells mobilize towards the perivascular zone, triggering the reactivity of brain endothelial cells, leading to BBB leakage and the massive infiltration of immune cells [8,9]. Furthermore, a persistent neuroinflammatory state is associated with alterations in the expression of ECM receptors and ligands, basal lamina proteins, membrane receptors, and tight junctions of endothelial cells [10,11,12]. Moreover, inflammatory signaling can induce endothelial cell dysfunction and de-differentiation, promoting the transition from endothelial to mesenchymal configuration [13,14]. Though the aforementioned molecular alterations foreshadow the considerable disruption in BBB function and disease progression, imaging tools for diagnosing and targeting these changes in real time are still scarce.

The technology of the in vivo selection of phage-displayed peptides is a valuable tool for the identification of specific ligands in tissue sites under pathological conditions such as cancer and neuroinflammation [15,16,17,18]. In vivo selections mainly target the vascular system, presenting both disease-altered targets and unaffected molecules. Although, other useful methodologies for target identification and validation exist and have been tested in neurodegenerative and inflammatory conditions [19], such as high-throughput screening, which is advantageous when little is known of a target, allowing the assay and screening of a large number of biological effectors and modulators against designated and exclusive targets [20]. The screening and elimination of common peptide ligands has resulted in the discovery of peptide binding inflammatory specific targets [17] using a phage-displayed library of 12 peptides to select those bound to blood vessels in the CNS parenchyma of rats with experimental autoimmune encephalomyelitis (EAE) [17]. EAE is a model of neuroinflammation that mimics significant neuropathological aspects of multiple sclerosis [17,21]. BBB disruption is one of the EAE characteristics, occurring due to the abnormal expression of endothelial cell proteins, which results in perivascular infiltration of peripheral immune cells and the development of focal inflammatory lesions [22,23]. Among several phage-displayed peptides selected in the EAE animal model, we have identified specific peptides capable of labeling the human endothelial cell line HCMEC/D3 under proinflammatory conditions in in vivo binding studies. One of these peptides expressed by the phage clone 88 showed high specific affinity under inflammatory conditions with the endothelial cell line under IL-1β exposure [17]. The purpose of the present study is to validate the interaction of the peptide-88 with its possible targets expressed under inflammatory conditions by using nano-scale liquid chromatographic tandem MS (nLC-MS) of the cross-linked peptide to target proteins.

## 2. Results

### 2.1. Phage 88 Crosslinks with Target Proteins of a Human BBB In Vitro Model

**Figure 1:**
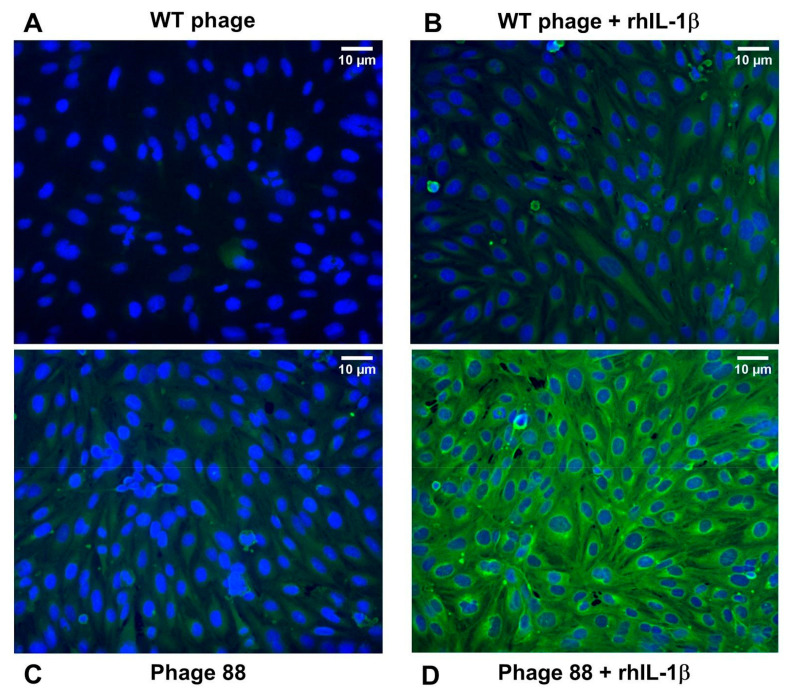
Crosslinking of phage 88 and target proteins on hCMEC/D3 cells. Sulfo-SBED labeled phage 88 or WT were crosslinked with protein targets on the hCMEC/D3 cells under rhIL1-β simulating proinflammatory conditions or in a resting state. (**A**) cells without stimulus of rhIL-1β incubated with WT phage; (**B**) cells stimulated with rhIL-1β and incubated with WT phage; (**C**) cells without stimulus of rhIL-1β and incubated with phage clone 88; and (**D**) cells stimulated with rhIL-1β and incubated with phage-peptide-88. The crosslinking is detected by labeling of Sulfo-SBED with streptavidin-DyLight 488 (green). Nuclei stained with DAPI are shown in blue. Phage photographs are representative images from two independent experiments (10×). Bar = 10 μm.

As previously reported, phage 88 binds to blood vessels in the CNS of EAE rats and can be used to indicate potential molecular alterations in the BBB due to inflammation [17]. To verify the specific binding of phage 88 to an in vitro human endothelial cell model of BBB [24], we used the HCMEC/D3 cell line and applied a crosslinking strategy to visualize their binding under inflammatory conditions. The crosslinking reaction used the trifunctional crosslinker Sulfo-SBED (Figure 1A). In this reaction, the Sulfo-SBED amine group was ligated to the phage expressing peptide-88 or the WT phage (Figure 1B). Cells previously treated with IL-1β to simulate inflammatory conditions or cells in a resting state without stimulation were incubated with 10^12^ pfu of each phage for peptide interaction with the target proteins. A UV-activated aryl azide bond was formed between the crosslinker and the target proteins on the cell surface of endothelial cells. After the separation of the phages, the Sulfo-SBED moiety attached to the target protein was visualized with streptavidin-DyLight 488 (green) (Figure 1). The immunofluorescence images of the WT phage incubated with endothelial cells showed no interaction under inflammatory or resting conditions (Figure 1A,B). Remarkably, phage 88 displayed a high affinity for target proteins in hCMEC/D3 cells stimulated with IL-1β (Figure 1D), whereas no specific binding was observed with non-stimulated cells (Figure 1C). The crosslinked proteins with phage 88 showed an extracellular distribution at the cell surface, but also exhibited a perinuclear binding inside the cells (Figure 1D). Mean fluorescent intensities (green Sulfo-SBED with streptavidin-DyLight 488/blue DAPI) were obtained for each image: WT phage, 0.181 arbitrary units (a.u.); WT phage + rhIL-1β, 0.184 a.u.; phage 88, 0.513 a.u.; phage 88 + rhIL-1β, 1.275 a.u., reflecting the interactions observed in the images. No statistical comparison was made between the groups due to the low n number.

### 2.2. Identification and Selection of Target Proteins Crosslinked with Phage 88

To identify the target proteins that specifically crosslink to the phage 88 in the HCMEC/D3 cells under inflammatory conditions, we reduced the disulfide bond of the Sulfo-SBED to separate the phage particles and cleave the interactions between the phage and protein targets. These proteins remain bound to the rest of the Sulfo-SBED molecule and are separated through biotin–streptavidin interaction.

The collected proteins were analyzed using nLC-MS/MS and quantified considering the area under the curve calculated for each detected peptide. The nLC-MS/MS analysis allowed the detection of 801 peptides assigned to 1269 proteins in experiment 1 and 843 peptides assigned to 1117 proteins in experiment 2 at 1% FPR (Table S1). A total of 112 proteins were included as potential candidates to be further analyzed, considering only proteins with a minimum of three peptides, and identified in both replicates.

For statistical analysis, the data of each biological experimental duplicate were analyzed independently and then combined. Among the 112 proteins, the binding occurrence ratio under IL-1β and control conditions between phage 88 and WT phage was determined. In order to obtain proteins with more affinity for phage 88 than WT phage, values higher than WT signal plus WT noise (1/3 of signal) were required, so proteins with a Ph88/WT ratio higher than 1.33 in the IL-1β-stimulated or control group were retained, filtering out proteins that bind unspecifically to the cells preferably associated with WT phage. For each biological experiment, the results of IL-1β-stimulated cells were normalized by its control, dividing the Ph88/WT ratio of IL-1β-stimulated cells by the Ph88/WT ratio of control cells to remove proteins not related to inflammation, obtaining the IL-1β/control ratio which allow comparison between biological replicates. Finally, the proteins showing a similar tendency between the two biological experimental duplicates were considered (Table S1).

**Figure 2:**
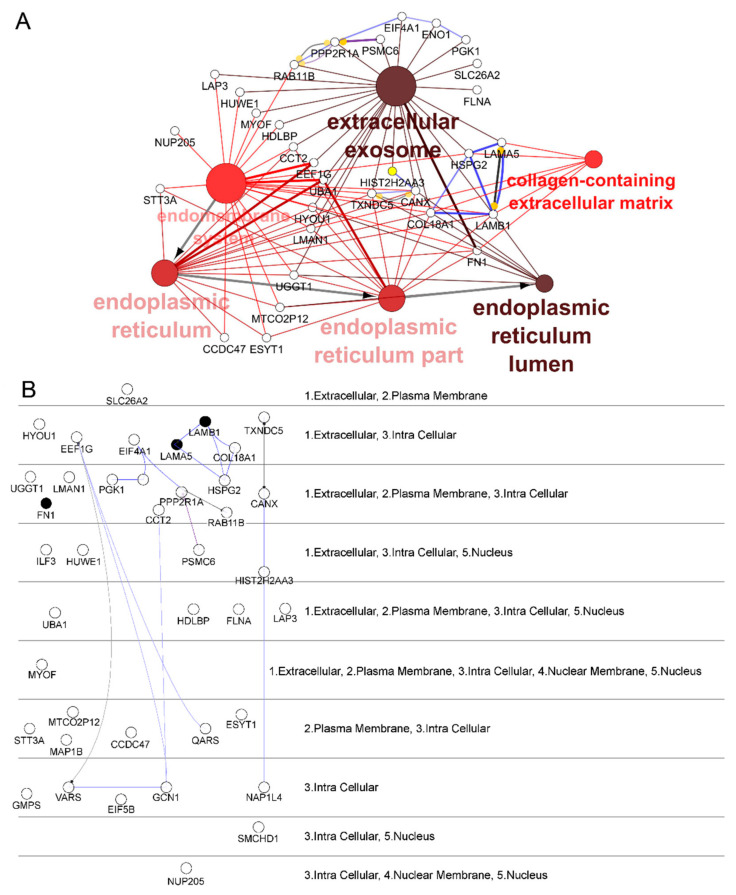
CC-GO functional annotations of candidate proteins identified by nLC-MS analysis. Proteins filtered by a Ph88/WT ratio higher than 1.33 and normalized by the control group were selected. (**A**) The associated CC-GO terms were extracted, enriched and grouped on a functional network, with ClueGO-Cytoscape. The larger the node, the greater the number of mapped genes; the darker the color, the lower the value of p (greater statistical significance). The embedded STRING network, representing the functional association of mapped genes, was created with CluePedia-Cytoscape. Color edge: black represents “reaction”, blue represents “binding”, and purple represents “catalysis”; width edge was scaled according to the confidence score. The mapped protein and its associated terms is shown, highlighting candidate target proteins based on their subcellular location. (**B**) Location of mapped proteins split according to extracellular (1), plasmatic membrane (2), intracellular (3), nuclear membrane (4) or nuclear (5) presence. Cerebral layout of STRING network, showing only interactions between genes, without nodes enrichment. Filled circles represent proteins selected for further experiments.

Forty proteins passed the criteria and were analyzed by enrichment analyses of functional terms with ClueGO v2.5.3-Cytoscape v3.7.1. Due to our interest in the subcellular location of identified proteins, cellular component gene ontology (CC-GO) was selected as the functional annotation. The associated terms were selected, applying the options “term fusion” and “significance selection,” and were grouped based on the kappa score. After *p*-value significance selection (*p*-value < 0,05), 31 proteins associated with six representative terms remained (Figure 2A). The CC-GO terms were merged in three groups: extracellular exosome, endomembrane system, and endoplasmic reticulum lumen, enclosing the other terms: collagen-containing ECM, endoplasmic reticulum and endoplasmic reticulum part. The CC-GO grouped network reflects the relationships between the terms based on the similarity of their associated proteins (Figure 2). The STRING network, showing the functional and physical interactions among mapped proteins, was created with CluePedia v1.5.3-Cytoscape v3.7.1, and is shown inside the term grouped network. This protein network has shown laminin subunits α5 and β1 binding with the intracellular proteins collagen alpha-1(XVIII) chain (COL18A1) and basement membrane-specific heparan sulfate proteoglycan core protein (HSPG2), as denoted by the blue edge (Figure 2A), as well as the localization of laminin subunits and fibronectin, in filled circles, at both the extracellular and intracellular regions (Figure 2B).

The selection of potential target proteins was made according to their function as structural proteins of the cellular membrane or their appearance in the ECM (gene ontology annotation), considering they are the first proteins that encounter the peptide-88. Further, published reports of the role of these proteins in neuroinflammation and BBB were considered. The reports were examined via a search in the PubMed database using the following keywords: BBB and neuroinflammation.

Fibronectin and laminin (both subunits α5 and β1) were selected as potential target proteins, as they presented a Ph88/WT ratio higher than 1.33 and significant differences between hCMEC/D3 cells under inflammatory (IL-1β treatment) or control conditions. Several reports have demonstrated the presence of laminin and fibronectin in the basement membrane of brain endothelial cells [25]. Both proteins play an essential role in promoting cellular adhesion to ECM and in the formation of tight junctions, evidenced by enhanced transepithelial/transendothelial electrical resistance (TEER) in in vitro models [26]. Indeed, research using recently developed in vitro models such as the human brain endothelial microvessel-on-a-chip [27], or the human BBB on-a-chip in a high-throughput microfluidic device [28], which closely replicate the BBB microenvironment, have revealed the importance of fibronectin and laminin in human brain endothelial cell adhesion to the ECM. These proteins are present in relevant subcellular locations, including extracellular and intracellular regions (Figure 2B). Furthermore, laminins interact between them and with other components of the basement membrane and intracellular proteins, including COL18A1 and HSPG2, as shown in the STRING network (Figure 2A).

### 2.3. Peptide-88 Binds to the Laminin Subunit Beta-1

We evaluated the binding interaction of fibronectin and laminin (both subunits α5 and β1) directly to peptide-88 through a solid-phase microplate protein-binding assay. We also tested the protein flotillin-1, as a negative control considering that this protein did not exhibit any affinity with the phage 88 neither in IL-1β stimulation or control conditions.

**Figure 3:**
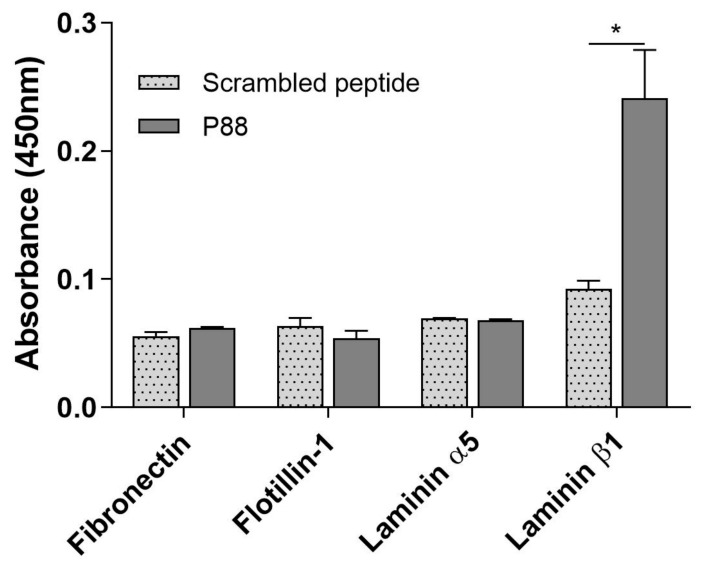
Evaluation of peptide-88 binding to selected human proteins. A solid-phase microplate protein-binding assay was developed to identify the target protein of peptide-88. The scrambled peptide comprises twelve amino acids forming a linear peptide biotinylated at the N-terminal that exhibited no binding to control cells or IL-1β treated cells. The scrambled peptide and peptide-88 were used at 500 μg/mL. The absorbance was determined in a microplate reader at 450 nm. A blank was also included for each evaluated protein that consisted of PBS instead of the peptide as an internal control. Statistical differences were determined by Student’s *t*-test (* *p* < 0.05, *n* = 2).

All plates coated with the recombinant proteins exhibited a significant increase in the absorbance according to the serial dilutions with the primary antibody as the positive control. In contrast, the scrambled peptide displayed similar absorbance values to the blank control in each protein coating plate. These results indicate no unspecific signal due to reagents or to the binding of peptide-88 to the recombinant proteins because of their size or biochemical properties. Notably, peptide-88 did not show significant binding to fibronectin, flotillin-1, or laminin subunit α5, but presented a significative binding to the laminin subunit β1, evidenced by an increased absorbance in comparison to the scrambled peptide (*p* < 0.05) (Figure 3).

### 2.4. Binding Mode of Peptide-88 to Laminin 511

**Figure 4:**
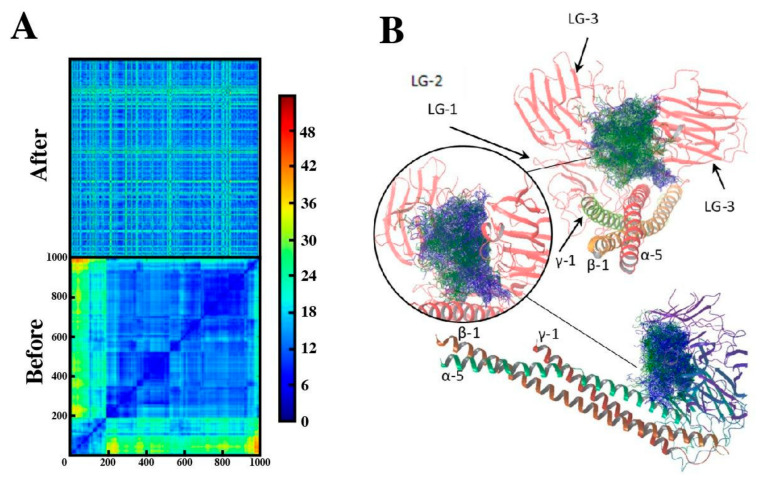
Clustering of peptide-88 poses performed by atomic RMSD comparison. (**A**) The symmetrical distance matrix illustrates atomic RMSD comparison of the 1000 poses of peptide-88 found by molecular docking. On the diagonal line, the RMSD is zero because the poses are compared with themselves. Matrix of peptide-88 poses organized before (top panel) and after (bottom panel) clustering process. The input order is kept on the diagonal; accordingly, the clusters are now visible as squares on the line. The sidebar represents the RMSD atomic distance scale in Å. (**B**). Significant clusters 16 and 20 are represented by blue and green lines, respectively.

In the present study, we found that peptide-88 binds to laminin subunit β1. However, the lack of laminin crystallographic structures does not allow a comprehensive conformational analysis to explore the binding modes of peptide-88 with this protein. For this reason, we chose the laminin-511 isoform (composed by subunits α5-β1-γ1), whose 3D structure is the only available to study how the peptide-88 interacts at a structural level. We applied a systematic pipeline that includes conformational sampling, massive docking simulations, and clustering analysis. Takizawa et al. described the molecular basis of the interaction between laminin-511 and the α6-β1 integrin [29]. Therefore, we included the α6-β1 integrin binding site in our computational study as well as the laminin subunit β1 domain as putative binding sites of peptide-88. Thus, the docking was targeted to residues 2676–3293 from laminin subunit α5, residues 1716–1786 from laminin subunit β1, and residues 1535–1604 from laminin subunit γ1. At the end of the massive docking simulations, we obtained 1000 different conformations of peptide-88 interacting with laminin-511. These conformers were clustered, and 25 clusters were obtained. The average population was 40 ± 64.03, which implies that significant clusters are those whose population is greater than 168.06 (average + (2 × standard deviation)). Thus, clusters 16 and 20 were significant, with populations of 174 and 246 peptide-88 conformers, respectively (Table S2). Figure 4 shows both significant clusters for peptide-88 and the RMSD clustering process. Previous studies have shown that the integrin binding to the laminin-511 is evidenced in the LG1-3 chain of the α-5 subunit and the tail of the carboxyl-terminal of the γ-1 chain, which is in accordance with the results obtained for both clusters. Contacts with subunit β1 were also evidenced for cluster 16, where laminin subunit β1 residue Arg1769 is interacting with the peptide-88 through hydrogen bonds with residues Thr2 and Pro3; Leu1772 is also interacting with peptide-88 residues Met4 and Met5 through hydrophobic contacts (Table S3). These interactions with laminin subunit β1 confirm our experimental findings and allow us to hypothesize a possible peptide-88 binding site in the entire laminin (α5-β1-γ1) protein. This hypothesis could not be experimentally confirmed as laminin-511 is not available as recombinant protein for non-competitive protein-binding assays.

**Figure 5:**
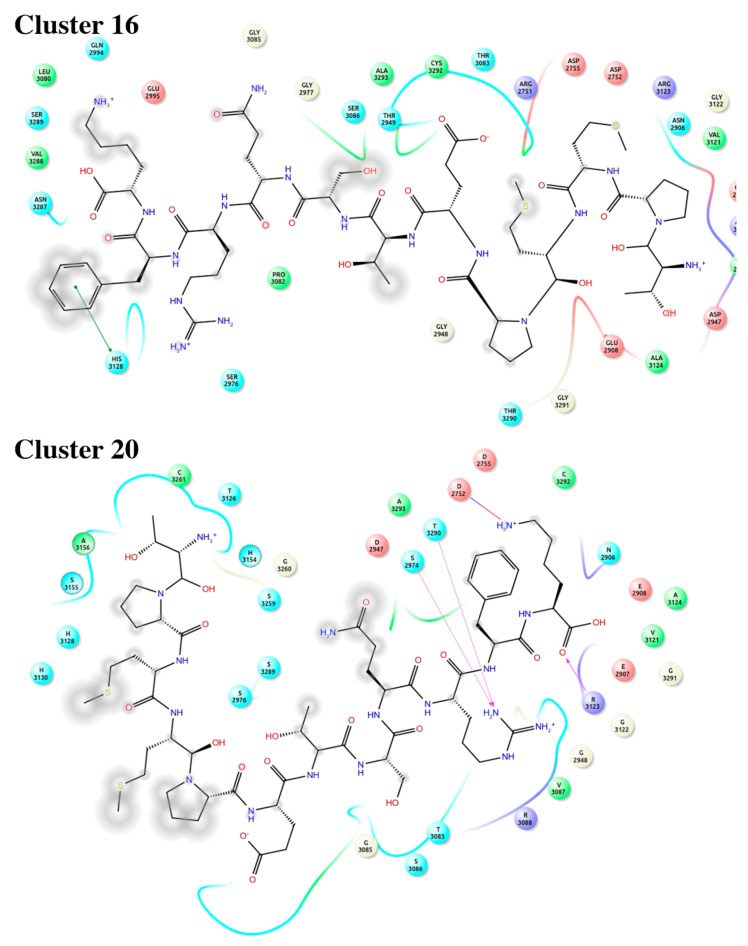
Interaction 2D diagram of the representative structure for both significant clusters 16 (C-16) and 20 (C-20) with laminin-511. Green circle: aromatic π–π interactions, Red circle: hydrophobic interactions, Blue circle: hydrogen bond.

Both significant clusters present a complex interaction network mainly composed of hydrogen bonds and hydrophobic contact. However, other types of contacts were identified, such as salt bridges, aromatic interaction π–π face-to-face, and edge to face (Tables S2–S6). Figure 5 shows the 2D-interaction diagram between the representative conformer of each cluster and laminin-511.

## 3. Discussion

During neuroinflammatory processes, it is frequently observed a differential expression profile in the proteins exhibited in the vascular endothelium that conforms to the BBB. The identification of these cellular markers is crucial for developing new strategies for earlier and real-time diagnosis. We previously identified an EAE-specific phage clone through a phage display approach [17]. The clone-denominated phage 88 displayed a conjugated peptide with high selectivity for neuroinflammatory conditions both in blood vessels in the CNS of EAE rats and in the HCMEC/D3 cell line, which is a validated in vitro endothelium model of BBB. In the present work, we applied a crosslinking strategy and MS analysis to isolate and identify the target proteins interacting with phage 88 on the HCMEC/D3 cells under inflammatory conditions.

Our proteomic data revealed that 40 proteins differentially marked the cells among inflammatory and control conditions. These proteins interact with phage 88 and not to wild type phage, indicating a higher affinity for peptide-88. Considering that the crosslinking occurred between a phage expressing peptide-88 and the endothelial cells by interacting with exposed proteins on the cell surface, the most likely site for the first interaction with target proteins is either at the extracellular side of the cell membrane or at the ECM. However, it is not possible to rule out that phage expressing peptide-88 proteins could be internalized with their target proteins and localized intracellularly, as we observed in the immunofluorescence images. Further, some target proteins, which interact with phage 88, could form complex networks of interaction with other proteins that can be detected in the MS analysis. Therefore, we focalized the analysis in extracellular proteins or associated cellular membrane, filtering the proteins with GO cellular component annotations including extracellular space and plasma membrane. Moreover, we filter target proteins with reported relevant neuroinflammatory effects on BBB, according to the PubMed database. The protein interaction network and subcellular localization, depicted in Figure 2, highlight the status of laminin subunits α5 and β1 connecting the extracellular region with intracellular components. Moreover, the laminin subunit β1 was found to be associated with the hippocampal neuronal perinuclear region [30,31].

Fibronectin, together with α5 and β1 subunits of laminin 511, appeared in our MS data analysis as potential candidate target proteins to be tested by affinity assays with the peptide-88. Remarkably, several reports suggest the involvement of these proteins in BBB function and neuroinflammation [32,33,34,35,36,37,38,39,40]. The MS results showed that the subunits of laminin 511 (α5 and β1) and fibronectin presented higher affinity to the phage clone expressing peptide-88 than the WT phage. Moreover, these proteins in the IL-1*β*-stimulated cells also showed a higher affinity for phage 88 than the same proteins in the control cells. Remarkably, laminin subunit α5 and β1 and fibronectin were also found in higher abundance in IL-1*β*-stimulated hCMEC/D3 cells than in non-stimulated cells, as those were probably over expressed under inflammatory conditions. Although flotillin-1 presented no significant association with phage 88 under inflammatory or normal conditions, several reports indicate a role in BBB and neuroinflammation [41,42]. For this reason, flotillin-1 was used as a negative control in our experiments.

The synthesized peptide-88, without conjugation with phage M13, was tested in a solid-phase microplate protein-binding assays with the recombinant human proteins of laminin (subunits α5 and β1), fibronectin and flotillin-1. The results showed that peptide-88 specifically binds to laminin subunit β1, but not to the other analyzed proteins. In addition, the scrambled peptide showed comparable absorbance values to the blank control in each assay, indicating no unspecific signal due to the binding of peptide-88 to the recombinant proteins because of their biochemical properties. Although we could not test the direct interaction of peptide-88 with the complete structure of laminin-511 in vitro, we modeled the peptide interaction with laminin-511 using a massive docking approach. A complex interaction network mainly composed by hydrogen bonds and hydrophobic contacts was found (Tables S2–S5). What stands out best is how laminin residues Arg1769, Arg2751, Ser2974, Thr3083, Arg3088, Arg3123, Ala3124, Thr3290, and Cys3292 interacts with peptide-88 through several hydrogen bonds. Important hydrophobic contacts were also identified, suggesting a strong peptide interaction with the laminin-511. We also identified interactions with the reported residues that form part of the α6-β1 integrin binding site [29]. The results obtained have allowed us to identify the complex interaction network of peptide-88 with laminin-511. This type of massive molecular docking analysis has been successfully used in protein–ligand interaction analysis [43,44] and is a tool that allows us to identify key residues in a fast and effective way, to later corroborate by site-directed mutagenesis the mode and site of interaction. For this reason, we suggest performing this type of validation in subsequent studies.

These results suggest that peptide-88 is a potential biomarker of laminin-511, which is differentially expressed on BBB under inflammatory conditions. Laminin-511 (α5-β1-γ1) is expressed in brain microvascular endothelial cells (BMECs), but it has been less studied than laminin-411 (α4-β1-γ1), which is also present in those cells [35]. Nevertheless, laminin-511 has been reported to improve the BBB integrity by stabilizing the tight junctions and reducing leukocyte extravasation under neuroinflammatory conditions [45,46]. A higher expression of laminin-511 (α5, β1 and γ1) inhibits T lymphocyte migration, avoiding the interactions between lymphocytes α6β1 integrin and laminin-411 (α4, β1 and γ1 chains) [47].

Cell interactions with the ECM are fundamental to promote cell adhesion, neurite outgrowth, axon guidance, the differentiation of neural progenitor cells and angiogenesis. Laminin, as a structural component of the basement membrane, contributes to the preservation of the physical barrier of the gliovascular layer formed by the end-feet of astrocytes and the mature BBB [48]. It has been reported that the lack of laminin α2 subunit expression on astrocytes alters the stability of the BBB, inducing leakage into the brain parenchyma [48]. This also modifies the morphology of the astrocytes which become hypertrophic, affecting the adequate polarization of aquaporin 4 (AQP4) channels. AQP4 channels are important for the regulation of water volume in the brain under physiological and pathological conditions [49,50]. Another study showed that a decrease in the expression of the laminin receptor in astrocytes leads to an increase in the expression of vascular endothelial growth factor (VEGF) and disruption of the dystrophin–AQP4 complex, which would then induce vasogenic edema formation and subsequent endothelial laminin over-expression [51]. Recent efforts have been implemented to treat brain vasogenic edema through targeting the mechanism of calmodulin-mediated cell-surface localization of AQP4 [52], and through pharmacological interventions on AQP4 with the FDA-approved drug trifluoperazine [53].

The identification of bioactive peptides which mimic ECM proteins has been of particular interest. For instance, it was reported that the laminin-derived Ile-Lys-Val-Ala-Val sequence, designed as a bio-functional peptide, improves stem cell behavior for cell replacement therapy in traumatic brain injury [54]. Additionally, laminin subunit β1 is localized in the perinuclear region of the cytoplasm, and laminin subunit α5 could be localized in the nucleus [55]. Notably, phage 88 is not exclusively located at the cell surface, but also is distributed around the nucleus. This observation suggests an internalization of the Ph88–laminin complex and possibly explains the presence of intracellular proteins in the data analysis.

Many peptide-targeting agents have been identified by in vivo phage display; however, the characterization of these targets is very scarce, especially in neuroinflammatory processes that alter the BBB. Nonetheless, some studies have identified peptide ligands to vascular structures. An in vitro study using phage display screening on the endothelial protein vascular adhesion protein-1 (VAP-1) identified a specific ligand peptide, denominated Siglec-9, with potential imaging biomarker properties [56]. Another study tested an in vivo phage display screening strategy to identify a specific peptide ligand to pancreatic tumor vasculature [57]. The authors used a functional proteomics approach to identify the potential target protein, similar to the approach that we used here. 

We have identified the bioactive peptide-88 for cerebral vascular endothelium interacting specifically at proinflammatory conditions through phage display screening. Using a functional proteomic analysis, we were able to identify its possible target and interaction through molecular simulation. Peptide-88 is capable of labeling the ECM protein laminin 511, highlighting its possible importance as a biomarker of the changes that occur in the BBB structure under certain pathological circumstances. Our study proposes a route for identifying and characterizing potential biomarker peptides similar to peptide-88, thus allowing a more precise tracing of the molecular bases of the neuroinflammatory process. In particular, this approach can help to explore BBB changes during the early stages of neurodegenerative diseases. This finding may provide a more precise pathological detection and early diagnosis, improving the current treatment practice from an advanced late-stage to a more timely intervention.

Under physiological conditions, the ECM proteins covering the endothelial cell surface serve both as a physical barrier and as a defense element for the BBB. The endothelial cells are constantly exposed to the mechanical forces generated by blood flow. This hemodynamic stimulus activates mechanosensors together with signaling pathways, and gene and protein expression, which impact endothelial cells’ function and morphology [58]. Changes in the glycocalyx of human brain-like endothelial cells due to flow have been recently reported in a microfluidic lab-on-a-chip model [59]. In this study, flow was shown to increase barrier properties through the induction of important endothelial and BBB genes. In addition, the flow upregulated ECM genes and rendered the endothelial cell surface more negatively charged and richer in lectin binding sites. Additionally, in blood outgrowth endothelial cells (BOECs), flow has been shown to induce the reorganization of laminin–integrin networks within the endothelial basement membrane [60]. Although static models, such as the one used in our work, still offer valuable information about BBB properties and interactions, future studies will benefit from more advanced models based on microfluidic systems, as they are able to better replicate the dynamic conditions of brain endothelial cells.

The brain endothelial cells constituting the BBB have strong interactions with other cells of the NVU such as pericytes (both share a common basal membrane), and astrocytes (end-feet cover brain vessels). Correspondingly, interactions of brain endothelial cells with the ECM are crucial to promote the development, maintenance, and regulation of BBB functions [59]. As the model we used in our study has the limitation of a 2D cellular growth, we suggest that future examinations of the relationships between laminin, brain endothelial cells (together with other NVU cells), and neuroinflammation, use the most complex 3D and microfluidic experimental models. Therefore, studies based in co-cultures of brain endothelial cells with pericytes and astrocytes, can provide more accurate information about the physiological environment of the BBB. In addition, studies based on 3D cultures allow structures to be organized and supported by an ECM, which facilitates the cell–cell interactions via transmembrane receptors and helps modulate cyto-skeleton organization and gene expression, even of the basal lamina proteins [61]. Another advantage of 3D microfluidic cell cultures involves the possibility of using advanced optical imaging. A recent study using TY10 human brain endothelial cells cultured on a 3D microfluidic microvessel-on-a-chip model, was able to obtain images from quantitative 3D live fluorescence imaging using spinning disk confocal, lattice light sheet microscopy (LLSM) and high resolution electron microscopy [27].

## 4. Materials and Methods

### 4.1. Amplification and Purification of Phages

Wild type (WT) phage consists of M13 phage without conjugated peptide, and phage 88 is comprised of M13 phage conjugated with the peptide-88 (TPMMPETSQRFK) displayed as a fusion of the coat protein pIII (New England Biolabs, Ipswich, England). Phages were amplified in 10 mL of log growth phase of the *E. coli* strain ER2738 (New England Biolabs, Ipswich, England) for 4 h at 37 °C under agitation (200 rpm). After phage amplification, bacteria were eliminated by centrifugation at 8000 rpm for 10 min at 4 °C, and the supernatant containing the viral particles was removed and purified with polyethylene glycol (PEG)/NaCl (20% (*w*/*v*) PEG-8000, 2.5 M NaCl) for 1 h at 4 °C. After centrifugation at 14,000 rpm for 10 min at 4 °C, phage pellets were dissolved in tris buffered saline (TBS)/0.02% NaN_3_. 

### 4.2. hCMEC/D3 Cell Culture

The human brain endothelial cell line hCMEC/D3, an immortalized suitable in vitro model for BBB, was kindly provided by Dr. Pierre-Olivier Couraud, Institut Cochin, Paris. hCMEC/D3 was cultured following the protocol previously reported [17,18]. Cells were plated on rat tail collagen I (Life Technologies, Carlsbad, CA, USA) pre-coated Petri dishes (Life Technologies, USA), or Lab-Tek chamber slides (Dutscher, Bernolsheim, France). The cells were seeded at a density of 25,000 cells per cm^2^ and grown until the cells reached 90 to 95% confluence. 

Cells were cultured at 37 °C, 5% CO_2_ atmosphere in EBM-2 basal medium (Lonza) containing 5% fetal calf serum (FCS), 10-mM HEPES (Dutscher, Bernolsheim, France), 1 ng/mL of basic fibroblast growth factor (bFGF) (Sigma-Aldrich, St. Louis, MO, USA), hydrocortisone (1.4 µM), ascorbic acid (5 µg/mL), chemically defined lipid concentrate (CONC, Life Technologies, USA), and 1% antibiotics (penicillin–streptomycin). The medium was supplemented with 10 mM lithium chloride and replaced every 3 to 4 days. The cells became completely confluent within 4 days. All experiments were performed on cultures of confluent cells between passage 29 and 35. Periodically, the culture was tested for mycoplasma contamination using the MycoProbe^®^ Mycoplasma Detection Kit (Catalog # CUL001B, R&D Systems, Abingdon, UK). 

### 4.3. Crosslinking of Phage 88 and Target Proteins on hCMEC/D3 Cells 

**Figure 6:**
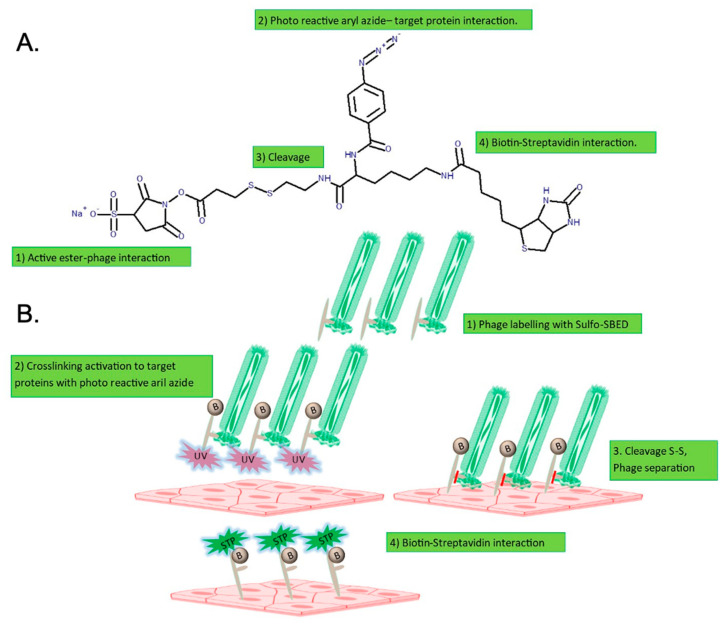
Experimental strategy for detection and isolation of target proteins of peptide-88. (**A**) Molecular structure of sulfo-SBED. (**B**) Crosslinking phage peptide ligand with hCMEC/D3 targets. Biotin (B), streptavidin (STP), ultraviolet (UV).

The crosslinking between phage 88 and target proteins on hCMEC/D3 cells was performed using a Sulfo-N-hydroxysuccinimidyl-2-(6-[biotinamido]-2-(p-azidobenzamido)-hexanoamido)ethyl-1,3′-dithioproprionate (Sulfo-SBED) crosslinker reagent (Thermo Scientific, Rockford, IL, USA). Sulfo-SBED is a trifunctional crosslinking reagent containing three moieties: a sulfonated N-hydroxysuccinimide (Sulfo-NHS) active ester with a disulfide bond; a photoreactive aryl azide; and a biotin group (Figure 6A).

The crosslinking strategy followed four stages (Figure 6B): (1) the Sulfo-NHS ester reacts with primary amines on the phage surface at pH 7–9, resulting in a covalent amine bond; (2) peptide expressed on phage 88 interacts with its protein targets in cells and upon UV photoactivation, the aryl azide reacts with amine groups, crosslinking the cell surface proteins targets; (3) the moiety with the disulfide bond is cleaved by reduction with Tris 2-carboxyethyl phosphine hydrochloride (TCEP) in glycine solution, allowing for the separation of the phage particles and the dissociation of peptide–protein target interaction between phage 88 and protein targets on the cell surface; (4) the moiety with aryl azide remains attached to the proteins targets linked to the biotin arm, allowing one to separate or to detect the proteins recognized by the phage 88 by using biotin–streptavidin interaction.

The phages were labeled with Sulfo-SBED as previously described [62,63]. Briefly, the phage 88 and WT phage at 10^12^ pfu in 1 mL of PBS were incubated with 50 µL of Sulfo-SBED 70 mM, for 1 h at 4 °C in the dark. The labeled phages were purified to discard unbound Sulfo-SBED by precipitation in PEG/NaCl according to the protocol described above and resuspended in PBS. 

hCMEC/D3 cells were stimulated with 20 ng/mL of recombinant human interleukin 1 beta (rhIL-1β) or an equivalent volume of the vehicle for 2 h. Subsequently, cells were incubated with 100 µL of phage 88 or WT phage pre-labeled with Sulfo-SBED for 1 h at 4 °C in the dark to obtain four experimental conditions: (1) cells without stimulus of rhIL-1β incubated with WT phage; (2) cells stimulated with rhIL-1β and incubated with WT phage; (3) cells without stimulus of rhIL-1β and incubated with phage clone 88; and (4) cells stimulated with rhIL-1β and incubated with phage-peptide-88. The crosslinking assays were made in two independent experiments, both for immunofluorescence detection and for target protein separation.

### 4.4. Immunohistochemistry 

hCMEC/D3 cells from all experimental conditions and seeded on Lab-Tek chamber slides were fixed with 4% formaldehyde for 30 min and washed twice with PBS.

After fixation, cells were treated for 20 min with 0.1 N HCl and saturated with 3% goat serum in PBS for 1 h. The unbound phages were removed by three washes of 5 min with 0.1% Tween-20 in PBS. The chamber slides were placed on ice, and the crosslinking between phages and the target proteins was induced by UV exposure with an 8 Watt 365 nm lamp for 15 min. The phages bound to their target proteins were eluted by cleaving the crosslinking with 100 µL of TCEP in the glycine solution at pH 2.5 for 10 min. Cells were washed twice for 5 min with glycine solution and then washed with PBS for 5 min. The samples were incubated with streptavidin–DyLight 488 for 1 h in the dark at room temperature and washed three times for 5 min with PBS, followed by incubation with 4′, 6-diamidino-2-phenylindole (DAPI) for 5 min and 2 further washes for 10 min with Tris-HCl. After removing the chamber, the cells were covered with VECTASHIELD^®^ Mounting Medium (Vector, Laboratories, Burlingame, CA, USA) and cover glass. The cells were observed and photographed using an epifluorescence microscope (Nikon 90i). The images were observed using 10× objectives with a numerical aperture (NA) value of 0.25. For each treatment, only one field of view, which covered almost the entire cell-populated well, was acquired. The images were processed using the program ImageJ v1.52p (NIH, Bethesda, MD, USA). For each image, the background signal was subtracted using the following commands in ImageJ: “Process”, then “Subtract Background”, and next, a rolling ball radius value of 20 pixels was used. The green (Sulfo-SBED with streptavidin-DyLight 488) and blue (DAPI) fluorescence intensities were measured using the following commands in ImageJ: “Analyze”, and then “Measure”. The calculated mean value of green fluorescence was divided by the mean value of blue fluorescence to control for the number of cells in the image.

### 4.5. Isolation of Target Proteins of Phage 88 Crosslinked on hCMEC/D3 Cells

Cells submitted to all experimental conditions were lysed in 1 mL of non-denaturing lysis buffer (50 mM Tris HCl pH 7.5, 150 mM NaCl (TBS), with 1% Triton X-100 and 5 mM EDTA) with a mixture of mammalian protease inhibitors, and then transferred to button-tubes. The lysate was incubated for 20 min with 40 µL of Dynal M280 streptavidin beads (Thermo Fisher Scientific, Rockford, IL, USA). The proteins were retained by placing the tube in a DYNAL MPC-1 magnet for 5 min. The supernatant was removed, and the beads were washed with 1% Triton X-100 in PBS three times. The protein-magnetic beads were suspended in 200 µL of denaturation buffer (1 M Tris-HCl pH 6.8, 20% SDS, glycerol and blue bromophenol) and boiled in a water bath for 5 min to cleave the biotin–streptavidin interaction. The samples were centrifuged at 14,000 rpm. The magnetic beads pellet was discarded, and the supernatant with the target proteins was collected. The protein concentration was determined according to the bicinchoninic acid (BCA) protein assay (Thermo Fisher Scientific, Rockford, IL, USA).

### 4.6. Sample Preparation

Proteins obtained from the crosslinking among phages and hCMEC/D3 cells were solubilized in Laemmli buffer and loaded onto a 10% acrylamide SDS-PAGE. After colloidal blue staining, each lane was cut and subsequently divided into smaller pieces. Gel slices were destained in 25 mM ammonium bicarbonate (NH_4_HCO_3_)/50% acetonitrile (ACN), rinsed twice in ultrapure water and shrunk in ACN for 10 min. After ACN removal, gel slices were dried at room temperature, covered with the trypsin solution (10 ng/µL in 50 mM NH_4_HCO_3_), rehydrated at 4 °C for 10 min, and finally incubated overnight at 37 °C. Slices were incubated with 50 mM NH_4_HCO_3_ for 15 min at room temperature with rotary shaking. The supernatant was collected, and an H_2_O/ACN/HCOOH (47.5:47.5:5) extraction solution was added onto gel slices for 15 min. The extraction step was repeated twice. All supernatants were pooled and concentrated in a vacuum centrifuge to a final volume of 45 µL. Digests were finally acidified by adding 1.5 µL of formic acid (5% *v*/*v*) and stored at −20 °C.

### 4.7. LC-MS/MS

Peptides were subjected to nano-scale liquid chromatography coupled to tandem mass spectrometry (nLC-MS/MS) on an Ultimate 3000 nanoLC system (Dionex) coupled to a nanospray LTQ-Orbitrap XL mass spectrometer (Thermo Finnigan, San Jose, CA, USA). An amount of 10 µL of peptide digests were loaded onto a C18 PepMapTM trap column (300 µm-inner diameter × 5 mm, LC Packings) at a flow rate of 30 µL/min. The peptides were eluted from the trap column onto an analytical C18 Pep-Map column (75 mm id × 15 cm, LC Packings) with a 5–40% linear gradient of solvent B in 90 min (solvent A was 0.1% formic acid in 5% ACN, and solvent B was 0.1% formic acid in 80% ACN). The separation flow rate was set at 200 nL/min. The MS operated in positive ion mode at a 1.8-kV needle voltage and a 49-V capillary voltage. Data were acquired in a data-dependent mode, alternating an FTMS scan survey over the range *m*/*z* 300–1700 and six ion trap MS/MS scans with collision-induced dissociation (CID) as the activation mode. MS/MS spectra were acquired using a 3 *m*/*z* units ion isolation window and normalized collision energy of 35%. Mono-charged ions and unassigned charge-state ions were rejected from fragmentation. Dynamic exclusion duration was set to 30 s.

### 4.8. Protein Identification and Quantification

Protein identification was performed with Proteome Discoverer 1.3 using the SEQUEST search engine (Thermo Fisher Scientific, Rockford, IL, USA.) against the *Homo sapiens* Reference Proteome Set (Uniprot version 2013-01; 70,425 entries). Spectra from peptides higher than 5000 Da or lower than 350 Da were rejected. Search parameters were as follows: mass accuracy of the monoisotopic peptide precursor and peptide fragments was set to 100 ppm and 0.8 Da, respectively. Only b- and y-ions were considered for mass calculation. Oxidation of methionines (+16 Da) was considered as variable modification. Two missed trypsin cleavages were allowed and only “high confidence” peptides were retained, corresponding to a 1% false-positive rate (FPR) at peptide level. Results were provided as a “grouped” listing to present the minimal number of proteins covering all detected peptides. A quantitative analysis was performed through Progenesis LC-MS 4.0. The area under the peak of each of the detected peptide ions (MS1) was integrated and the area of peptides were summed to give the area of the protein, allowing the comparison among samples in a given biological experiment. Thereby, the intensity of the protein was reconstructed by summing its specific peptides. Data normalization was carried out, considering the total intensity of all the proteins present in the sample. The relative normalization was carried out by dividing the intensity of each identified protein by the total intensity of all proteins recognized in each sample. Proteins with at least 3 quantified peptides in both cross-linked replicated assays lists were considered (experiment 1 and experiment 2). Data for each experiment were analyzed separately: a second normalization was carried out by separately calculating the ratio of phage 88 (Ph88) against the WT phage (WT) under the two different conditions, IL1-β-treated or control (hCMEC/D3) cells, known as the Ph88/WT ratio: (1) Ph88 +IL-1β/WT +IL-1β; (2) Ph88 -IL-1β/WT -IL-1β (control). 

Proteins with a P88/WT ratio higher than 1.33 in any experimental condition (IL-1β or control) were considered. In order to remove the proteins linked in a treatment-unspecific way, the results were normalized by the P88/WT ratio of control cells (hCMEC/D3), obtaining IL-1β/control fold: (P88 IL-1β/WT IL-1β)/(P88 control/WT control). In addition, proteins with a similar P88/WT ratio between treatment and control, and an IL-1β/control fold of 1 or higher were also considered. Finally, the results of both biological replicates were combined, selecting those proteins with a P88/WT ratio tendency similar between experiment 1 and 2, and obtaining the mean and standard error of the mean (SEM) of the P88/WT ratio of each experimental condition and the IL-1β/control fold. (Table S1).

### 4.9. Bioinformatic Analysis of Proteomic Data

The list of proteins resulting from the processing data obtained in proteomics was mapped to UniProtKB protein entries based on accession, and protein annotations were extracted, including gene ontology (GO) terms for cellular component (CC), involvement in disease and tissue specificity (UniProtKB/Swiss-Prot 2018_04-April 25, 2018) (“UniProtKB/Swiss-Prot 2018_04,” n.d.).

The selected proteins were integrated and analyzed using ClueGO v2.5.3., and CluePedia v1.5.3. (Integrative Cancer Immunology, Paris, France) [64,65] Cytoscape v3.7.1 plugins (U.S. National Institute of General Medical Sciences (NIGMS), Bethesda, MD, USA) [64]. CluGO functional enrichment analysis of the 40 considered proteins was performed using GO: CC (GO_CellularComponent-EBI-UniProt-GOA_31.01.2019), 19,032 genes, *Homo sapiens*, all evidence codes used, GO level: 2–6 to classify proteins by subcellular location. Options as “Term fusion” and “GO term restrictions” (number. of gene/term ≥ 6) were applied to reduce the related term redundancy. CC-GO terms with *p*-value less than 0.05 were accepted. A two-sided hypergeometric statistical test was conducted to determine the principal cellular component associated with target proteins. The Bonferroni method was used to correct the probability value. The enriched terms were grouped based on the kappa score (kappa score threshold = 0.4, merge redundant groups with >50.0% overlap). CluePedia was used to visualize the known functional and physics interactions, from STRING, between the GO terms and the associated proteins.

### 4.10. Evaluation of Peptide-88 Binding to Target Proteins

Solid-phase microplate protein-binding assays were performed to evaluate proteins that could potentially bind to the peptide-88, optimizing the conditions previously described [66]. The commercial recombinant proteins tested include fibronectin (ACFP4305B, R&D systems, Minneapolis, MN, USA), laminin α5 (H00003911-Q01, Novus Biologicals, Centennial, CO, USA), laminin β1 (NBP2-42385PEP, Novus Biologicals, Centennial, CO, USA), and flotillin-1 (H00010211-Q01, Novus Biologicals, Centennial, CO, USA) as a negative control. The peptide-88 (TPMMPETSQRFK, GenScript, Piscataway, NJ, USA) was synthesized conjugated to biotin at the N-Terminal to detect it with a streptavidin system.

A 96-well plate was coated with 4 μg/mL of recombinant protein at 4 °C overnight and blocked with 5% BSA/PBS for 2 h at room temperature. After washing, serial dilutions of biotinylated peptide-88 or primary antibody against the target proteins were added to the wells, followed by overnight incubation at 4 °C. The plate was incubated with streptavidin-HRP (1:200 diluted in 3% BSA/PBS; DY998, R&D systems, Minneapolis, MN, USA) for 30 min at room temperature and washed. Substrate reagent (DY999, R&D systems, Minneapolis, MN, USA) was added and incubated for 30 min at room temperature. The reaction was stopped (DY994, R&D systems, Minneapolis, MN, USA) and the absorbance was measured at 450 nm using a microplate reader. Wells with PBS instead of peptide-88 or primary antibody were used as negative controls. A biotinylated scrambled peptide (LPSTQPALPPNA, GenScript, Piscataway, NJ, USA) was also included in each assay to verify the binding specificity between the peptide-88 and target proteins.

### 4.11. Computational Studies

To study the molecular basis of the interaction of peptide-88 with the detected proteins (flotillin-1, fibronectin and laminin), we explored the protein data bank and found that only laminin had been resolved to date. Laminins are conformed by three chains, α, β, and γ, which assemble into a cross-shaped heterotrimer. In mammals, 11 laminin chains (α1–α5, β1–β3, and γ1–γ3) and 16 combinations of these have been identified [67]. To explore how peptide-88 interacts with the studied proteins, and due to the lack of crystallographic structures, we selected the laminin-511, conformed by α5β1γ1 subunits. The target protein (PDB code: 5XAU) and the peptide-88 were prepared using the Protein Preparation Wizard (Schrödinger Release 2019-1: Maestro, Schrödinger, LLC, New York, NY, USA, 2021. Academic Version). The evaluation of the interaction between peptide-88 with the laminin was carried out through a massive molecular docking methodology using the AutoDock Vina software (ADV, version 1.1.2, La Jolla, CA, USA) [68]. The dimensions of the used grid box area for the docking simulations were 70_x_, 48_y_, 106_z_ (Å^3^). An *in-house* script was used to run 100 molecular docking simulations, and the top-10 solutions of each run were taken according to the ADV scoring function [69]. This procedure assured an extensive conformational sampling that explored many of the possible conformations of peptide-88 within laminin. We obtained a total of 1000 poses to explore the most representative interaction conformations. To process and organize all 1000 poses, we used the Conformer Cluster script available in the Maestro software. This script builds a matrix using a measure of the pairwise distance between the 1000 poses. This measure was the root mean square displacement (RMSD) between pairs of corresponding atoms following optimal rigid-body superposition. The atomic RMSD was calculated considering the heavy atoms as well as the hydroxyl (-OH) and thiol (-SH) groups of the peptide-88 conformers analyzed. The average linkage method was used to cluster the peptide-88 poses. Significant conformational clusters, for which the populations depart by more than 2 standard deviations (σ) from the mean cluster population [44], were selected for further analysis by using the poseviewer_interactions.py script available at the Schrödinger suite.

### 4.12. Statistical Analysis

Statistical analysis was performed using GraphPad Prism 8.0 (GraphPad Software Inc., San Diego, CA, USA). Two biological replicates were used for immunofluorescence and protein-binding assays. Unless otherwise indicated, a *p-*value equal to or less than 0.05 was considered statistically significant for all analyses and not corrected for multiple comparisons. The data are presented as the mean ± standard error of the mean (SEM), if not stated otherwise.

## Data Availability

All data generated or analyzed during this study are included in this published article and its Supplementary Information files.

## Supplementary Materials

The following are available online at https://www.mdpi.com/article/10.3390/ijms22094725/s1.

## Abbreviations

ACN	acetonitrile
AMBIC	ammonium bicarbonate buffer
CID	collision-induced dissociation
DTT	dithiothreitol
IAA	iodoacetamide
MS	Mass Spectrometry
SDS-PAGE	sodium dodecyl sulfate polyacrylamide gel electrophoresis
TFA	Trifluoroacetic acid
Sulfo-SBED	Sulfo-N-hydroxysuccinimidyl-2-(6-[biotinamido]-2-(p-azido benzamido)-hexanoamido) ethyl-1,3′-dithioproprionate
