# Supplementary material for: Screening for Interacting Proteins with Peptide Biomarker of Blood–Brain Barrier Alteration under Inflammatory Conditions
Source: Int J Mol Sci. 2021 Apr 29;22(9):4725. doi: 10.3390/ijms22094725 (PMC8124558; doi:10.3390/ijms22094725)
Supplement: Supplementary file 1 [file ijms-22-04725-s001.zip › Table S2, S3, S4, S5, S6.pdf]

Table S2. Total of conformational clusters obtained by the clustering process of 1000 poses.

No. Cluster	Population
1	1
2	3
3	1
4	25
5	2
6	24
7	46
8	9
9	6
10	25
11	23
12	14
13	7
14	2
15	161
**16**	**174**
17	4
18	55
19	110
**20**	**246**
21	39
22	11
23	7
24	2
25	3
Average	40
Standard Deviation	64.03

Table S3 Interaction matrix between the residues of the protein (*y* axis) and the residues of the peptide 88 (*x* axis) for the significant cluster16. Each value in the table body correspond to the number of conformers which interact with the specific residues. Hydrogen bridges (HB) are in blue, Hydrophobic interactions (HI) are in orange, Saline bridges (SB) are in green, Aromatic interaction π-π face to face (FF) are in re and Aromatic interaction π-π edge to face (EF) are in pink.

	2(THR)	3(PRO)	4(MET)	5(MET)	6(PRO)	7(GLU)	8(THR)	9(SER)	10(GLN)	11(ARG)	12(PHE)	13(LYS)
ARG 1769	2	2										
LEU 1772			2	1								
ALA 2714	2											
ARG 2718	1 6	8	5	2								
ARG 2720							1					
GLU 2721	3 1	6	5	10	4		7					
LEU 2722	3	9	1	2								
ALA 2724		1	5	11	1		5					
GLN 2725	5	2	4	14	4	2						
PRO 2750			11	1								
ARG 2751	1 1	1 5	6 19	3 5	10 9	7 6 1	2	3				
ASP 2752	1 9 8	23	**61**	5	3							
ASP 2755	**19** 5	19	30									
LEU 2756	1		13									
LEU 2905			5									
ASN 2906	3	5	23									
GLU 2907	10	22	3	2								
GLU 2908	41	28	**51**	25	2							
VAL 2909			1		1							
TYR 2945	1	3	3	1								
ASP 2947	3 12	9	28	20								
THR 2949		2	34	**68**	19	10	1					
PHE 2951	5	2	11	3	3							
VAL 2973			3	5	1							
SER 2974					4	**20**	6	2	6			
TYR 2975						12		1	1			
SER 2976						14		4				2
GLN 2994					1		2		3	1	12	9
GLU 2995					1		2		1 7	4 3	19	6 8 17
PRO 3069	7	2	6	2								
PRO 3070	4	5	9									
ASP 3071	2 2 2	1	2									
ARG 3078											1	
ARG 3079											6	1
LEU 3080											9	4
PHE 3081						2	16		1	4	4	2
PRO 3082						1	5		6	19	27	13
THR 3083	2	5	6	2 6	7	10 9	3 16	11	6 6	1 8	16	1 8
GLY 3085								1	2		1	
SER 3086						5		3	2	1		
ARG 3088	1 1	1	13	25	14	13 1 20	6					
THR 3109	1		2	1								
ARG 3123	14 10	21	3			1 1						
ALA 3124	4 35	40	16	2		1 1						
THR 3126				1	1	2	3		9	16	29	16

HIS 3128									2	8	**3** **9** 22	13
HIS 3130											**1**	
HIS 3154										2	3 **5**	1
ALA 3156											1 8	3 13
GLN 3157											3	3
TYR 3203	5											
SER 3259											2	7
CYS 3261		4	10									
ASN 3287												3
SER 3289							1	7				1
THR 3290					2	**26**	5	6	4			
CYS 3292	11 10	**32** 16	1 13			1						
ALA 3293		3	6	35	30	4 10	3 13	5	8 30	3 12	36	2 23

Table S4. Interaction matrix between the residues of the protein (*y* axis) and the types of interactions (*x* axis) for the significant cluster-16. Each value in the table body coespond to the number of conformers which have the specific type of interaction. Hydrogen bridges (HB) are in blue, Hydrophobic interactions (HI) are in orange, Saline bridges (SB) are in green, Aromatic interaction π-π face to face (FF) are in re and Aromatic interaction π-π edge to face (EF) are in pink.

	HB	SB	FF	EF	HI
ARG 1769	4				
LEU 1772					3
ALA 2714					2
ARG 2718	1				16
ARG 2720					1
GLU 2721		3			28
LEU 2722					15
ALA 2724					23
GLN 2725	5				26
PRO 2750					12
ARG 2751	28	6			39
ASP 2752	1	9			93
ASP 2755		**19**			48
LEU 2756					14
LEU 2905					5
ASN 2906					31
GLU 2907					37
GLU 2908					**110**
VAL 2909					2
TYR 2945					8
ASP 2947		3			69
THR 2949					**110**
PHE 2951					20
VAL 2973					9
SER 2974	**38**				
TYR 2975	14				
SER 2976	20				
GLN 2994					28
GLU 2995	7	12			47
PRO 3069					16
PRO 3070					17
ASP 3071	3	2			4
ARG 3078					1
ARG 3079					6
LEU 3080					14
PHE 3081					33
PRO 3082					67
THR 3083	32				73
GLY 3085	4				
SER 3086	11				
ARG 3088	15	1			79
THR 3109					4
ARG 3123	15	1			34
ALA 3124	5				85
THR 3126					73
HIS 3128			**3**	**9**	44
HIS 3130				1	

HIS 3154	6			5	
ALA 3156	4				18
GLN 3157					5
TYR 3203					5
SER 3259	9				
CYS 3261					14
ASN 3287	3				
SER 3289	9				
THR 3290	**43**				
CYS 3292	**45**				36
ALA 3293	25				**133**

Table S5. Interaction matrix between the residues of the protein (y axis) and the residues of the peptide 88 (x axis) for the significant cluster-20. Each value in the table body correspond to the number of conformers which interact with the specific residues. Hydrogen bridges (HB) are in blue, Hydrophobic interactions (HI) are in orange, Saline bridges (SB) are in green, Aromatic interaction π-π face to face (FF) are in re and Aromatic interaction π-π edge to face (EF) are in pink.

	2(THR)	3(PRO)	4(MET)	5(MET)	6(PRO)	7(GLU)	8(THR)	9(SER)	10(GLN)	11(ARG)	12(PHE)	13(LYS)
ARG 2718											2	
ARG 2720							5				1	1
GLU 2721	1			1		14	9		1		6	2 7
LEU 2722											2	
ALA 2724					2	7	7		1		8	2
GLN 2725					5	5	9				7	2
LYS 2732												3
PRO 2750											14	
ARG 2751				1	7	6 5 2	13	40	18 10	10 12	6 26	7 7
ASP 2752						1 2	2		24	14 29	66	1 42 21
ALA 2754											2	
ASP 2755										7 2	37	18 30
LEU 2756											12	
LEU 2905											11	
ASN 2906										1	25	6
GLU 2907											20	20
GLU 2908									3	1 8	79	3 39
VAL 2909											3	2
TYR 2945											1	
ASP 2947									3	1 8	42	5 10
THR 2949		2			8	7	7		30	31	32	22
PHE 2951											3	2
VAL 2973							2					
SER 2974	3	1				9	10	12	6	1		3
TYR 2975	4					8	1	6	6			
SER 2976						2		8	4			
GLN 2994	5	19	5	2		1	1					1
GLU 2995	2 4 10	7	16	4	4	2 1	1			1	1	1 1
PRO 3069											3	3
PRO 3070	1		1	2		1					2	4
ASP 3071												1
ARG 3078	7	3	1									
ARG 3079	1	2	9	2								
LEU 3080	7	5	10	4	2							1
PHE 3081	6	20	8	6	20	9	17		1		1	2
PRO 3082	10	20	22	28	35	10	8			1		1
THR 3083	1 2	5	5 13	24	4 33	10 22	5 18	9	8 4	4	3	1 1
GLY 3085						4			1			1
SER 3086	1		1			4	5	4	2			
ARG 3088	3	4	4			1	1 15	5	6 13	3 16	15	23 5
ARG 3123									1		1 14	38 11
ALA 3124							1			5	40	38 67
THR 3126	11	34	24	23	13	8	13				4	
HIS 3128	12	2	23	11	3	1					3 1 3	
HIS 3154	4		1									
ALA 3156	3 12	8	5	2	1							
GLN 3157	2		3	1								

ASP 3158	1											
SER 3159	1											
PRO 3201		1	1	1							5	1
TYR 3256	1											
SER 3259	10	3	2	1	1							
CYS 3261	1	3	2		2						16	9
ASN 3287	5					1						
SER 3289	4					3	1	3				
THR 3290	5					15	7	8	11			2
CYS 3292	1		2				1	2	10	1 1	9 16	9 20
ALA 3293	3 16	24	1 33	38	1 30	8 23	1 23		1 33	12	17	7

Table S6. Interaction matrix between the residues of the protein (*y* axis) and the types of interactions (*x* axis) for the significant cluster-20. Each value in the table body correspond to the number of conformers which have the specific type of interaction. Hydrogen bridges (HB) are in blue, Hydrophobic interactions (HI) are in orange, Saline bridges (SB) are in green, Aromatic interaction π-π face to face (FF) are in re and Aromatic interaction π-π edge to face (EF) are in pink.

	HB	SB	FF	EF	HI
ARG 2718					2
ARG 2720					6
GLU 2721		2			36
LEU 2722					2
ALA 2724					27
GLN 2725					27
LYS 2732	3				
PRO 2750					14
ARG 2751	**88**	5			56
ASP 2752	2	**55**			**130**
ALA 2754					2
ASP 2755		25			65
LEU 2756					12
LEU 2905					11
ASN 2906					32
GLU 2907					40
GLU 2908		4			122
VAL 2909	5				
TYR 2945					1
ASP 2947		6			63
THR 2949					119
PHE 2951					5
VAL 2973					2
SER 2974	44				
TYR 2975	20				5
SER 2976	14				
GLN 2994					34
GLU 2995	6	5			43
PRO 3069					6
PRO 3070					10
ASP 3071					1
ARG 3078					11
ARG 3079					14
LEU 3080					29
PHE 3081					85
PRO 3082					110
THR 3083	42				114
GLY 3085	6				
SER 3086	17				
ARG 3088	38				76
ARG 3123	40				25
ALA 3124	38				112
THR 3126					122
HIS 3128			**1**	**2**	53
HIS 3154	5				
ALA 3156	3				27
GLN 3157					6
ASP 3158	1				

SER 3159	1				
PRO 3201					9
TYR 3256					1
SER 3259	17				
CYS 3261					32
ASN 3287	6				
SER 3289	11				
THR 3290	47				
CYS 3292	32				39
ALA 3293	15				**181**
